# Editorial: Mental Health and Positive Youth Development in Sport and Physical Activity Contexts

**DOI:** 10.3389/fpsyg.2021.752369

**Published:** 2021-09-15

**Authors:** Luis Calmeiro, Pedro Teques, Antonio Rosado, Mauro Virgílio Gomes de Barros

**Affiliations:** ^1^Division of Sport and Exercise Sciences, Abertay University, Dundee, United Kingdom; ^2^Faculty of Medicine, Institute of Environmental Health, University of Lisbon, Lisbon, Portugal; ^3^Polytechnic Institute of Maia, Maia, Portugal; ^4^Faculty of Human Movement, Lisbon, Portugal; ^5^University of Pernambuco, Escola Superior de Educacão Fisica, Recife, Brazil

**Keywords:** adolescents, coaching, quality of life, health interventions, youth sport

Participation in sport and physical activity has been associated with a variety of positive consequences to young people's physical, psychological, social, and spiritual health (Bouchard et al., 2012). Broadly defined, physical activity is thought to contribute to the development of internal and external health assets (Morgan and Ziglio, 2007), which allows participants to transfer skills to other contexts of life. For example, sports may help young people building a set of beliefs, skills, attributes, and knowledge leading to a healthy and productive life. Competence, confidence, character, compassion, and a sense of connection in young people are dimensions of healthy development that can be nurtured through sport and physical activity environments and applied to other domains (Lerner et al., 2009; Jones et al., 2011).

However, as a more specific form of physical activity, sport can also provide an environment where athletes experience pressure to reach elite level or win at all costs. This can lead to maladaptive patterns of cognitions, emotions, and behaviours that negatively impact mental health (Hughes and Leavey, 2012). For example, the prevalence of depression and anxiety in elite athletes has been shown to be at least as high as in the general population (Wolanin et al., 2015).

The purpose of this Research Topic is to gather a body of evidence that explores the topic of the positive and negative impact of sport and physical activity participation in young people's development and the protective role it may have in their well-being. We have gathered the contributions of 32 authors divided in two opinion papers and four original research contributions.

Bateman et al. present a reflection on how sport can foster positive development of young people and the fundamental role coaches have in that process. The authors have identified some of the problems of youth development through sport, emphasising that negative experiences may result from the overly competitive-driven culture that predominates in youth sport, and the coaches' lack of knowledge concerning how to intervene to promote positive youth development actively and purposefully. Therefore, the authors argue for the need for mandatory integration of positive youth development in coaching education in Australian sport. The authors also note the lack of large-scale research on positive youth development in Australian sport and suggest a research agenda to support claims concerning the benefits of increasing coach education on positive youth development.

Martins et al. provide a thought-provoking insight into the role of sport in positive youth development and mental health within the prison systems. This setting is widely understudied which is unfortunate. Indeed, sport programmes in this context may help young people develop a range of transferable skills that can be useful in other domains of life, contributing to a more efficient reintegration in society. Hence, from applied and research perspectives they suggest adopting an assets-based approach to design sport interventions and a Realist Evaluation Framework to assess the mechanisms through which outcomes are achieved within the specificity of the prison system context.

Concerning the research papers, two focused on physical activity and exercise, and two focused on competitive sport. Bandeira et al. evaluate the effects of a school-based lifestyle intervention on health-related quality of life using a cluster-randomised controlled trial. The intervention included (a) teacher training on physical activity, sedentary behaviour, and nutrition, (b) environmental improvements, and (c) educational strategies. The authors did not find significant differences between intervention and control groups, but they have provided a few recommendations to inform researchers when designing school-based interventions. Specifically, the authors emphasise the need for multi-arm interventions and the development and application of strategies that are compatible to each dimension of health-related quality of life.

Wu et al. studied (a) the mediation role of exercise value cognition and (b) the moderation role of only-child status on the relationship between family function and exercise behaviour of college students. The authors found that exercise behaviour was positively associated with family function and exercise value cognition. Moreover, family function significantly predicted exercise behaviour directly and indirectly through exercise value cognition. Finally, this mediating effect was moderated by only-child status so that exercise value cognition plays a greater mediating role between family function and exercise behaviour in the only-child group. This study suggests that strategies aiming at improving family function (e.g., quality of interactions) where the value of exercise is more efficiently communicated are likely to be efficient. Such communication seems to be facilitated in only-child families.

Liang et al. applied a progressive muscle relaxation intervention, twice a week for 1 month, to a group of track and field college student-athletes. Compared to a control group, the authors observed significant differences in pre-competitive state self-confidence in the 2 weeks following the beginning of the intervention and in sport performance after the intervention. Self-confidence differences disappeared and somatic and cognitive anxiety trends differed as the competition approached illustrating the dynamic nature of these psychological processes.

Finally, Carreres-Ponsoda et al. report the results of an intervention conducted in competitive football that aimed at improving personal and social responsibility, prosocial behaviours, and self-efficacy of young footballers. This intervention used the personal and social responsibility (TPSR) model as a guiding framework and consisted of an initial 3-month period of training of coaches on TPSR, followed by a 6-month implementation period. This implementation consisted of three sessions per week, alongside a series of expert-led seminars for athletes. Using a pre- and post-test design and self-report methods the authors observed an increase of personal and social responsibility, prosocial behaviours, and self-efficacy in the experimental group compared to the control group. This intervention demonstrates TPSR constitutes a pedagogical model that relies on a nurturing coach-athlete relationship to develop essential personal and relational values.

Taken together these articles pinpoint the diverse range of sport and physical activity contexts within which mental health and positive youth development can be targeted to improve young people's quality of life. By emphasising current different international perspectives, these articles demonstrate the maturation and advances in the area in recent years and identify some current gaps. It is our wish that this topic of research enhances the exploration of new paths in research and intervention in the relationship between Mental Health, Positive Youth Development and Sport/Physical Activity.

## Author Contributions

LC wrote the first draft. PT, AR, and MB reviewed and contributed to subsequent drafts. All authors read and agreed with the final version of the manuscript.

## Conflict of Interest

The authors declare that the research was conducted in the absence of any commercial or financial relationships that could be construed as a potential conflict of interest.

## Publisher's Note

All claims expressed in this article are solely those of the authors and do not necessarily represent those of their affiliated organizations, or those of the publisher, the editors and the reviewers. Any product that may be evaluated in this article, or claim that may be made by its manufacturer, is not guaranteed or endorsed by the publisher.

## References

[B1] Bouchard C. Blair S. N. Haskell W. L. (2012). Physical Activity and Health. Champaign, IL: Human Kinetics.

[B2] Hughes L. Leavey G. (2012). Setting the bar: athletes and vulnerability to mental illness. Br. J. Psychiatry 200, 95–96. 10.1192/bjp.bp.111.095976 22297587

[B3] Jones M. I. Dunn J. G. Holt N. L. Sullivan P. J. Bloom G. A. (2011). Exploring the'5Cs' of positive youth development in sport. J. Sport Behav. 34, 250–267.

[B4] Lerner J. V. Phelps E. Forman Y. Bowers E. P. (2009). Positive youth development, in Handbook of Adolescent Psychology: Vol. 1. Individual Bases of Adolescent Development, 3rd Edn. eds Lerner R. M. Steinberg L. (Hoboken, NJ: Wiley), 524–558.

[B5] Morgan A. Ziglio E. (2007). Revitalising the evidence base for public health: an assets model. Promotion Educ. 14(2_suppl), 17–22. 10.1177/10253823070140020701x 17685075

[B6] Wolanin A. Gross M. Hong E. (2015). Depression in athletes: prevalence and risk factors. Curr. Sports Med. Rep. 14, 56–60. 10.1249/JSR.0000000000000123 25574886

